# Cross-Talk between Gut Microbiota and Heart via the Routes of Metabolite and Immunity

**DOI:** 10.1155/2018/6458094

**Published:** 2018-06-03

**Authors:** Jin Bu, Zhaohui Wang

**Affiliations:** Department of Geriatrics, Union Hospital, Tongji Medical College, Huazhong University of Science and Technology, Wuhan 430022, China

## Abstract

Considering the prevalence of cardiovascular disease (CVD), significant interest has been focused on the gut microbiota-heart interaction because the gut microbiota has been recognized as a barometer of human health. Dysbiosis, characterized by changes in the gut microbiota in CVD, has been reported in cardiovascular pathologies, such as atherosclerosis, hypertension, and heart failure. Conversely, gut microbiota-derived metabolites, such as trimethylamine/trimethylamine *N*-oxide (TMA/TMAO), can impact host physiology. Further, bacterial dysbiosis can disturb gut immunity, which increases the risk of acute arterial events. Moreover, studies of germ-free mice have provided evidence that microbiota diversity and the presence of a specific microbe in the gut can affect immune cells in hosts. Therefore, the changes in the composition of the gut microbiota can affect host metabolism and immunity. Importantly, these effects are not only confined to the gut but also spreaded to distal organs. The purpose of the current review is to highlight the complex interplay between the microbiota and CVD via TMAO and different immune cells and discuss the roles of probiotics and nutrition interventions in modulating the intestinal microbiota as novel therapeutic targets of CVD.

## 1. Introduction

Paralleling the improvement of the social economy and aging population, cardiovascular disease (CVD) becomes the leading cause of death and disability worldwide. According to the American Heart Association, the overall rate of death attributable to CVD in 2013 was 222.9 per 100,000 Americans, accounting for 35% of deaths before the age of 75 years [1]. Cardiovascular health including health behaviors (e.g., healthy dietary pattern, appropriate energy balance, and nonsmoking) and health factors (e.g., optimal blood lipid level and blood pressure (BP)) has been recently defined as the primary goals. The role of the ecological system consisting of gut commensal bacteria in cardiovascular health becomes the focus of the current research.

In CVD, the ratio and abundance of the gut microbiota change and interventions using prebiotics, probiotics, and synbiotics share similar therapeutic efficacies in attenuating cardiac dysfunctions. Moreover, accumulating evidence indicates that bacterial dysbiosis increases cardiometabolic risks. It is likely that the gut microbiota substantially contributes to the global epidemic of CVD.

The microbiota in the gut, coevolving with the host, mainly colonizes in the colon. It can perform multiple functions, such as fermentation of nondigestible dietary substances, control of intestinal epithelial cell proliferation, and prevention from propagation of pathogenic microorganisms [2, 3]. Accumulating evidence also shows that manipulation of the composition of the gut microbiota affects host metabolism and immunity [4], whereas the effect is not only confined to the intestine but also spreaded to distal organs through different pathways [5, 6]. In this review, we aimed to discuss the compositional and functional changes in the gut microbiota in relation to CVD, determine the effects of the gut microbiota on CVD from the view of trimethylamine *N*-oxide (TMAO) and immune cells, and evaluate how gut interventions can lead to novel therapeutic targets for CVD.

## 2. Changes in the Composition of the Gut Microbiota in CVD

The development of high-throughput sequencing of nucleic acids (i.e., DNA and RNA) for taxonomic mapping allows the identification of the origins and composition of the microbiota [7]. After birth, the intestinal tract is colonized by nonpathogenic microorganisms and maintains a coexisting and symbiotic relationship with microbial ecology, which evolves over time and is susceptible to both exogenous and endogenous modifications. Generally, 35,000 species of the gut microbiota have been classified into five phyla (i.e., Bacteroidetes, Firmicutes, Actinobacteria, Proteobacteria, and Verrucomicrobia). The composition and ratio of the intestinal flora seem to change during the pathogenesis of CVD (Table 1).

The previous observation in patients of atherosclerosis (AS) that the abundances of *Veillonella* and *Streptococcus* in atherosclerotic plaques were correlated with their abundance in the oral cavity first suggested that the plaque microbiota may be partly derived from the oral cavity and/or the gut [8]. Furthermore, periodontal pathogens may in turn influence plaque composition and rupture, accompanying with increasing risks for coronary artery disease [9]. Interestingly, patients with symptomatic AS had a higher relative abundance of *Anaeroglobus* in the oral cavity than asymptomatic AS control [10]. Patients with symptomatic AS demonstrated enriched genus *Collinsella* in the gut compared to healthy controls; gut metagenome may be associated with the inflammatory status of the host [11]. Taken together, these data seem to suggest that the gut microbiome is more proinflammatory in patients with CVD [12]. Specially, large-scale clinical research on patients with coronary AS demonstrated that the abundances of Enterobacteriaceae and *Streptococcus* increased, while those of probiotics (*Clostridium*) decreased [13]. Patients with heart failure (HF) showed significantly decreased diversity of the intestinal microbiome and downregulated key intestinal bacterial groups [14]. Older patients had diminished proportions of Bacteroidetes and larger quantities of Proteobacteria and enriched *Lactobacillus* [15] compared to younger patients with HF.

Studies on APOE^−/−^ rats provided direct evidence that AS susceptibility within a host may be influenced by gut (cecal) microbial transplantation. Cecal microbial transplantation from AS-prone versus AS-resistant inbred strains of mice enhanced choline diet-dependent AS. The study also revealed that *Prevotella* was positively correlated with atherosclerotic plaque lesions [16]. On the 7th day after surgery for acute myocardial infarction (AMI) in a previous study, the abundance of the gut microbiota, such as the Synergistetes phylum and Lachnospiraceae family, significantly increased, paralleling gut barrier impairment [17]. Bacteria from the phyla Bacteroidetes and Firmicutes were prevalent in spontaneous hypertensive rats. These findings suggest that bacteria from the oral cavity and perhaps even the gut may correlate with disease progression of CVD.

## 3. Gut Microbiota-Derived Metabolite: TMAO and CVD

The gut microbiota can elicit effects on the host through bioactive metabolisms. Recent research studies have established that TMAO, an intestinal microbiota metabolite of choline and phosphatidylcholine, can increase the risk of incident major adverse cardiovascular events [18, 19]. Initially, Wang et al. found that the three metabolites of the dietary lipid phosphatidylcholine, namely, choline, TMAO, and betaine, predicted the risk for CVD in an independent large clinical cohort [5]. Then, they found that omnivorous human subjects produced more TMAO than vegans or vegetarians following the same ingestion. Furthermore, oral broad-spectrum antibiotics to suppress the intestinal microbiota can suppress detectable endogenous TMAO in both the plasma and urine, suggesting that the dietary status and specific bacterial taxa affect TMAO concentration. Thus, the gut microbiota plays a specific role in TMAO formation [6].

TMAO is derived from a diet containing choline through the digestion of gut flora which metabolizes choline to trimethylamine (TMA), a gas that is then absorbed into the circulation. TMA can be catalyzed as TMAO by FMO3, a key rate-limiting enzyme in the liver [20]. Specially, FMO3, as a direct FXR target gene, can be activated by bile acids to upregulate expression [20].

In clinical studies, patients who had major adverse cardiovascular events also had higher baseline levels of TMAO than those who did not. The TMAO levels were associated with a 3.4-fold increased mortality risk. TMAO was directly correlated with the severity of HF, independent of the brain natriuretic peptide level and glomerular filtration rate [21]. One to 3 days after acute MI, the circulating TMAO concentrations have been observed to rise. Specifically, the level of TMAO levels in acute MI was associated with prognosis, which predicted adverse outcome of all-cause mortality or reinfarction (death/MI) at 2 years [22].

In functional studies, although the TMAO levels did not directly affect the BP in rats, they prolonged the hypertensive effect of angiotensin II (Ang II) by affecting the structure of receptors and Ang II [23, 24]. Furthermore, TMAO could also exacerbate cardiac fibrosis and left ventricular (LV) adverse remodeling and dysfunction in a model of cardiac hypertrophy and heart failure [25]. It was found that 3,3-dimethyl-1-butanol (DMB), an inhibitor of TMA formation, can prevent cardiac inflammation and fibrosis in western diet- (WD-) induced cardiac dysfunction [26]. In addition, TMAO is associated with cardiovascular burden, such as glycemic control [27], BMI, and renal fibrosis [28].

Regarding mechanisms, the gut microbiota-driven TMA/FMO3/TMAO pathway is a key regulator of lipid metabolism and inflammation. For example, a recent study suggested that dietary supplementation with TMAO, carnitine, or choline reduced the reverse cholesterol transport (RCT) in mice. RCT can be mediated by either the classic biliary route or the nonbiliary transintestinal cholesterol excretion (TICE) pathway [29]. During lipid metabolism, (1) TMAO significantly increases the expression of ABCA1 and ABCG1 in the liver which helps cholesterol efflux to apoA1 as the cholesterol acceptor; (2) TMAO in the gut also markedly reduces the mRNA expression of NPC1L1, which transports cholesterol into the enterocyte from the gut lumen; (3) TMAO reduces the bile acid pool in the liver, which is associated with the classic RCT by reducing synthetic enzymes CYP7A1 and CYP27A1; and (4) TMAO also reduces the expression of ABCG5/8 in the TICE pathway. Therefore, these complex processes inhibit RCT [6]. During proinflammation, (1) TMAO can promote the expression of scavenger receptors (SRs) in the macrophage, such as CD36 and SR-A [5, 30]; (2) TMAO can also activate the well-known mitogen-activated protein kinase, extracellular signal-related kinase, and nuclear factor-*κ*B signaling cascade in primary human aortic endothelial cells and vascular smooth muscle cells [31]; (3) TMAO can also promote activation of PKC/NF-KB/VCAM-1, which accelerates endothelial dysfunction including decreased endothelial self-repair and increased monocyte adhesion [32]; (4) TMAO may be associated with macrophage polarization in WD-fed rats by increasing TNF-*α* and interleukin 1*β* (IL-1*β*) levels and decreasing anti-inflammatory factor IL-10 levels [23]; and (5) TMAO can significantly trigger oxidative stress and activate TXNIP-NLRP3 inflammasomes to release IL-1*β* and IL-18 in a dose- and time-dependent manner [33] in vitro. These studies in vivo and vitro pave the way for the clinical regulation of intestinal microorganisms and dietary interventions to prevent the formation of TMAO and improve CVD.

## 4. Gut Microbiota and Immunity in CVD

AS and resulting CVD involve inflammatory reactions in which both the innate and acquired immunities are involved, and anti-inflammatory therapy has received concerns [34]. The gut mucosa as one of the largest immunological active organs in the human body harbors several hundred trillions of bacteria, which are closely tied with the immune system, each influencing and being influenced by the other [35]. Therefore, whether gut microbes interact with CVD in terms of immunity remains unclear.

Recent research revealed that patients with chronic inflammatory bowel diseases, such as Crohn's disease and ulcerative colitis, were at an increased risk of acute arterial events [36], especially young patients [37], and the disease inflammatory state may be an independent risk factor for acute arterial events. Clinical studies and animal experiments have demonstrated that elevated plasma cholesterol attributed to WDs promoted CVD development. In the process, dietary cholesterol initiates intestinal inflammation in epithelial cells [38], disrupts the immune homeostasis, induces gut dysbiosis, and increases CD4+ and CD8+ cell infiltration in distal organs, such as the heart [26]. Chronic inflammation of the gut in cases of dysbiosis affects not only itself but also the systemic circulation and central or peripheral tissues. Therefore, we can conclude that the intervention of intestinal immunity may be another novel therapeutic target for the prevention of CVD [34].

### 4.1. Gut Microbiota and Immunity in AS

The relationship between inflammation and immune responses has been clarified by our understanding of innate and adaptive immunology. First, inflammation as a result of innate and adaptive immunology is produced from the start to the end in AS. Innate immunology responses are initiated by the body's recognition of the signature molecules DAMPs and PAMPs, which could be recognized by pattern recognition receptors (PRRs) [39]. Macrophage PRRs, such as SRs, CD36, and SR-A, internalize oxidation-specific epitopes (i.e., exposed phosphocholine, malondialdehyde, and oxidized cardiolipin). Gut microbe-derived TMAO could enforce the expression of the receptor of SR-A and CD36, which promotes the formation of foam cells whose accumulation within the subendothelium or neointima constitutes the first step in AS. However, the study that germ-free mice were as susceptible to AS as mice with symbiotic bacteria suggested that endogenous substances initiated the inflammation [40]. Endogenous substances, such as cholesterol crystals deposited in mature atherosclerotic lesions, induced inflammation by stimulating the caspase-1-activating NLRP3 inflammasome [41]. In vitro, TMAO can also activate the NLRP3 inflammasome. Meanwhile, the formation of foam cells and the activation of NLRP3 are both early events. Therefore, the intervention of the gut may be considered therapies for the early prevention of coronary AS.

Adaptive immune responses contribute to the development and complications of atherosclerotic lesions. For example, reduced or functionally impaired regulatory T cells (Treg) lead to an increased incidence of AS [42], and adoptive transfer of Treg into hypercholesterolemic mice reduces lesion development [43]. A study in Ldlr^−*/*−^ AS mice showed that a cholesterol-rich diet can impair the Treg, whereas reversal of a hypercholesterol diet could prevent loss of lesional Treg [44]. Interestingly, an oral anti-CD3 antibody induced Treg and inhibited the development of AS in mice [45]. These observations suggest that the gut intervention can regulate the function and quantity of the Treg for preventing atherosclerotic CVD. In addition, it has been established in germ-free mice or mice treated with antibiotics that intestinal microbes are associated with the differentiation of T cells [46, 47].

The role of natural killer T (NKT) cells represents a link between the innate and adaptive immune systems in response to AS, and numerous murine studies have shown that NKT cells are proatherogenic via the activation of their secreted cytokines, such as Th1, Th2, and Th17 cytokines [48]. Adult germ-free mice have increased absolute or relative levels of colonic NKT cells and are immature and hyporesponsive to *α*GalCer stimulation of splenic NKT cells. Neonatal germ-free mice can restore their NKT cell number and phenotype when exposed to intestinal microorganisms containing NKT cell antigens. This suggests that the gut microbiota can affect the function of the NKT cells in the organs. Although the direct association of the gut microbiota with AS remains obscure, these research studies provide a perspective that the intervention for the gut may affect the immune cells in CVD.

### 4.2. Gut Microbiota and Immunity in HF

HF is primarily a clinical diagnosis that develops secondary to either LV systolic or diastolic dysfunction, which is the final outcome of various CVDs [49]. HF with a preserved ejection fraction (EF) and HF with a reduced EF account for 50% of cases [50]. The levels of numerous proinflammatory cytokines, such as TNF-*α*, IL-1, IL-6, and IL-12, have been observed to be elevated in patients and in animal models with HF [51, 52]. Elevated circulating CD14+ monocyte counts 3 days after MI predict a failure to recover the LV systolic function at 3 months post-MI in patients [53]. Endotoxins, an important stimulus for cytokine production in patients with HF [54], can originate from disrupted intestines resulting from a reduced cardiac output, potentially leading to further exacerbations. An early study on intestinal microbes and HF displayed the perspective of intestinal permeability, that is, gut hypothesis. Whether manipulation of the gut microbiota can attenuate HF and improve long-term outcomes remains to be determined.

## 5. Gut Microbiota as a Potential Therapeutic Target in CVD

The experimental evidence and clinical observations prove the functional link between the intestinal microbiota and CVD, suggesting the theoretical basis to manipulating intestinal microbiota to prevent CVD. The gut microbiota consisting of trillions of commensal microorganisms can express enzymes capable of interacting and interfering with the nutrition and drug we consume, ultimately impacting each other.

First, the use of prebiotics, probiotics, and synbiotics, which is the easiest way to interfere with microbiota composition, had a similar efficacy in reducing LV dysfunctions in obese insulin-resistant rats [55]. For example, probiotics, as a kind of live beneficial bacteria, are administered to reestablish an appropriate intestinal balance (Table 2). In patients with CVDs, consumption of *Lactobacillus plantarum* (DSM 9843) increased the intestinal microbial diversity compared to a placebo [56]. Probiotic *Bifidobacterium animalis* subsp. *lactis* LKM512 could decrease the fecal TMA concentration and BMI in individuals [57]. Lab4 probiotic consortium plus *L. plantarum* CUL66 possessed anticholesterolemic capabilities in wild-type C57BL/6J mice fed with a high-fat diet. Probiotic *Enterobacter aerogenes* ZDY01 attenuated choline-induced TMAO levels via remodeling of the gut microbiota in mice [58]. Unfortunately, the treatment using probiotic *L. casei* Shirota in patients with metabolic syndrome [59] and VSL#3 in nonobese men during the consumption of a hypercaloric and high-fat diet cannot attenuate the production of TMAO [60]. Moreover, probiotics consisting of different strains may have various effects on CVD; thus, identifying the appropriate strains is essential for therapy. Functional genomics on the most closely related reference strains provided specific treatment strategies to restrain TMA producers and limit their proliferation [61].

Second, the effects of other components in diets can regulate the composition of the intestinal flora (Table 3). In APOE^−/−^ mice fed with a high-fat/cholesterol diet[62], plant sterol ester (PSE), which elevated the relative abundance of *Helicobacter*, Erysipelotrichaceae, and the genus *Roseburia* in the gut, is associated with reduced cholesterol levels, aortic plaques, and body fat. Oat *β*-glucan (OBG) can elevate butyrate levels and promote Verrucomicrobia population expansion. Thus, it appears to protect against high-fat/cholesterol-induced atherogenesis [63]. High-fiber diets can increase the prevalence of acetate-producing bacteria and improve the levels of *Bacteroides acidifaciens*, thereby preventing the development of hypertension and HF in hypertensive mice [64]. Inulin-type fructans (ITFs) can reverse endothelial dysfunction via increased NO-producing bacteria in APOE^−/−^ mice [65]. Vegetable/fruit juices promote weight loss, increase vasodilator NO levels, and decrease lipid oxidation in healthy individuals by decreasing the proportion of the phylum Firmicutes and Proteobacteria and increasing Bacteroidetes and Cyanobacteria in the stool [66]. The combination of *ω*-3 PUFAs and proanthocyanidins can provide cardiovascular benefits by maintaining the standard proportions of bacterial subgroups in the gut of a healthy rat [67]. Dietary allicin, a potent antimicrobial compound found in garlic, also reduces the transformation of TMAO from L-carnitine through impacting the gut microbiota in mice [68]. In addition, 3,3-DMB, a structural analog of choline prevalent in wine, olive oil, and grapeseed oil, inhibits TMA production from the gut microbes by inhibiting distinct microbial TMA [69]. Resveratrol, a natural phytoalexin with prebiotic benefits, has been found to attenuate TMAO-induced AS by decreasing plasma TMAO levels and increasing hepatic bile acid neosynthesis by changing the intestinal flora [70]. In addition, *Ganoderma lucidum* (*G. lucidum*), a medicinal mushroom used in traditional Chinese medicine, has been reported to have antiobesity properties, which are mediated by modulating the composition of the gut microbiota [71]. *G. lucidum* and its high-molecular-weight polysaccharides may be used as prebiotic agents to prevent gut dysbiosis and obesity-related metabolic disorders in mice fed with a high-fat diet [72].

Given the abovementioned intervention, the gut can regulate microbial community, which can further improve CVDs. When exposed to poison, such as acrolein [73], the gut increased the levels of intestinal *Coprococcus* and enhanced macrophage atherogenicity in atherosclerotic mice. Thus, gut microbiota remodeling plays a role in the development of CVDs and risks.

## 6. Concluding Remarks and Future Perspectives

The evidence from animal and human studies supports that gut microbiota is in correlation with cardiovascular disease. Unappreciated complexity and considerable diversity of the bacterial microbiome have been gradually uncovered via culture-independent methods. However, the direct relationship between gut microbiota and cardiovascular disease remains obscure. In addition, the diversity of microbiome enhanced the difficulty in identifying strains in correlation with disease state, which restricted therapeutic interventions for the exact target.

Apart from these restrictions, intestinal flora as a barometer of human health is a novel therapeutic target for preventing CVD. Further, tunable expression platforms for the prominent microbiome in which gene expression is controlled by a synthetic inducer may be a good tool. Larger randomized controlled studies of adequate sample size and duration and well-defined therapeutic schedules and endpoints are strongly advisable.

## Figures and Tables

**Table 1:** Summary of human trials studying the association between gut microbiome and cardiovascular disease.

Study	Aim	Method	Outcome	Number
Koren et al. [8]	To investigate the effect of oral or gut microbiota on the microbial composition of atherosclerotic plaques	qPCR, 16S rRNA	The abundances of *Veillonella* and *Streptococcus* in atherosclerotic plaques correlated with their abundance in the oral cavity	30 adults: 15 CVD and 15 healthy
Hyvärinen et al. [9]	To investigate the association between coronary artery disease and periodontal pathogens	qPCR	Levels of *A. actinomycetemcomitans* associated with increased risk for CAD	179 CAD, 166 ACS, 119 healthy
Fak et al. [10]	To elucidate the relationship between the oral microbiota composition and patients with asymptomatic and symptomatic atherosclerosis	16S rRNA	Higher relative abundance of the bacterial genus *Anaeroglobus* in symptomatic atherosclerosis	27 symptomatic AS, 35 asymptomatic AS, 30 healthy controls
Karlsson et al. [11]	To investigate whether the gut metagenome is associated with symptomatic atherosclerosis	MEDUSA	Genus *Collinsella* enriched in patients, *Eubacterium* and *Roseburia* enriched in controls	12 symptomatic AS, 13 healthy controls
Jie et al. [12]	To systematically examine the composition and functional capacity of the gut microbiome in relation to cardiovascular diseases	Shotgun sequencing	Increased abundance of Enterobacteriaceae and *Streptococcus* spp. in patients	218 CVD, 187 healthy controls
Luedde et al. [14]	To systematically investigate specific changes of the intestinal microbiome in HF patients	16S rRNA	Decreased diversity of the intestinal microbiome and downregulated key intestinal bacterial groups, such as *Blautia*, *Collinsella*, Erysipelotrichaceae, and uncl in HF patients	20 HF, 20 controls
Kamo et al. [15]	To investigate whether gut microbiota in HF is associated with aging	16S rRNA	Diminished proportions of Bacteroidetes, larger quantities of Proteobacteria, and enriched *Lactobacillus* in older patients with HF	12 HF patients younger than 60 years, 10 HF patients 60 years of age or older

CAD: coronary artery disease.

**Table 2:** Summary of the human randomized controlled trials analyzing the effect of probiotic supplementation on CVD.

Authors	Aim	Population	Main findings	Duration
Karlsson et al. [56]	To clarify the effect of *L. plantarum*(DSM 9843) on intestinal microbiota in patients with cardiovascular disease	16 males with atherosclerotic plaque	Increased bacterial diversity and decreased concentration of isovaleric acid and valeric acid	4 weeks
Matsumoto et al. [57]	To investigate the effect of probiotic *Bifidobacterium animalis* subsp. *lactis* LKM512 on colonic TMA and atherosclerosis-related makers in healthy subjects	27 healthy adults	Reduced fecal TMA concentration and BMI	12 weeks
Tripolt et al. [59]	To investigate the impact of *Lactobacillus casei* Shirota (LcS) on the formation of TMAO in subjects with metabolic syndrome	30 subjects with metabolic syndrome	Not affecting levels of TMAO	12 weeks
Boutagy et al. [60]	To investigate whether multistrain probiotic VSL#3 would attenuate the increase in fasting plasma concentrations of TMAO following a high-fat diet	Nonobese males	Not affecting levels of TMAO	2 weeks

**Table 3:** Nutrition intervention alters gut microbiota composition and improves CVD.

Nutrition intervention	Main findings	Alterations in gut microbiota composition
PSE (plant sterol ester)	Cholesterol and aortic plaque ↓	Erysipelotrichaceae ↑
OBG (oat *β*-glucan)	Cholesterol, aortic plaque, weight, and fat ↓	Butyrate levels and Verrucomicrobia ↑
High-fiber diet	Improve hypertension and heart failure	Acetate-producing bacteria ↑
ITF (inulin-type fructans)	Reverse endothelial dysfunction	Akkermansia ↑ Bacterial taxa ↓
Vegetable/fruit juices	Promote weight loss, increase vasodilator NO, and decrease lipid oxidation	Firmicutes/Proteobacteria ↓ Bacteroidetes ↑ Cyanobacteria ↑
*ω*-3 PUFAs and proanthocyanidins	Plasma cholesterol ↓	Maintains the standard proportions of bacterial subgroups and their function
Allicin	TMAO ↓	Clostridium ↑
DMB	TMAO ↓	Inhibit distinct microbial TMA lyases
Resveratrol	TMAO ↓	*Lactobacillus* and *Bifidobacterium* ↑
*Ganoderma lucidum* mycelium	Weight, inflammation, and insulin resistance ↓	Firmicutes-to-Bacteroidetes ratios and endotoxin-bearing Proteobacteria levels ↓
